# The Role of Adrenomedullin in the Renal NADPH Oxidase and (Pro)renin in Diabetic Mice

**DOI:** 10.1155/2013/134395

**Published:** 2013-07-15

**Authors:** Michio Hayashi, Akihiro Tojo, Tatsuo Shimosawa, Toshiro Fujita

**Affiliations:** ^1^Department of Internal Medicine, University of Tokyo, 7-3-1 Hongo, Bunkyo-ku, Tokyo 113-8655, Japan; ^2^Division of Nephrology and Endocrinology, Department of Internal Medicine, University of Tokyo, 7-3-1 Hongo, Bunkyo-ku, Tokyo 113-8655, Japan

## Abstract

Adrenomedullin has an antioxidative action and protects organs in various diseases. To clarify the role of adrenomedullin in diabetic nephropathy, we investigated the NADPH oxidase expression, renin-secreting granular cell (GC) hyperplasia, and glomerular matrix expansion in the streptozotocin (STZ)-induced diabetic adrenomedullin gene knockout (AMKO) mice compared with the STZ-diabetic wild mice at 10 weeks. The NADPH oxidase p47phox expression and lipid peroxidation products were enhanced in the glomeruli of the diabetic mice compared with that observed in the controls in both wild and AMKO mice. These changes were more obvious in the AMKO mice than in the wild mice. Glomerular mesangial matrix expansion was more severe in the diabetic AMKO mice than in the diabetic wild mice and exhibited a positive correlation with the degree of lipid peroxidation products in the glomeruli. Proteinuria was significantly higher in the diabetic AMKO mice than in the diabetic wild mice. The GC hyperplasia score and the renal prorenin expression were significantly increased in the diabetic AMKO mice than in the diabetic wild mice, and a positive correlation was observed with the NADPH oxidase expression in the macula densa. The endogenous adrenomedullin gene exhibits an antioxidant action via the inhibition of NADPH oxidase probably by suppressing the local renin-angiotensin system.

## 1. Introduction

Adrenomedullin is a potent vasodilating peptide that is upregulated in cardiovascular diseases to counteract the disease process with its diverse physiological actions including antioxidative stress actions [1–6]. The plasma concentration of adrenomedullin also increased in the diabetic patients, and hyperglycemia increases the production of adrenomedullin in the vasculature [7, 8]. The receptors for adrenomedullin are expressed in the kidneys, especially in the glomerulus and distal nephron, and the local action of adrenomedullin is increased in diabetic rats [9], thus suggesting that adrenomedullin may contribute to the dilatation of the glomerular capillary in the early phase of diabetic nephropathy. Although the organoprotective effects of adrenomedullin have been demonstrated in various cardiovascular diseases, the mechanisms underlying its renoprotection in diabetic nephropathy are still unclear.

Hyperglycemia accelerates the formation of advanced glycation end products (AGE), while also upregulating the protein kinase C (PKC) activity, accelerating the polyol pathway, and promoting sorbitol deposition [10]. These pathways are related to the increased oxidative stress, which plays a significant pathogenetic role in diabetic complications. In the kidneys of diabetic animals, the expression of NADPH oxidase and its oxidative products is observed to increase, and the suppression of NADPH oxidase ameliorates renal damage and endothelial dysfunction in diabetics [11–16]. Adrenomedullin has been demonstrated to possess an antioxidant action; however, the relationship between NADPH oxidase and adrenomedullin in the kidneys of diabetic nephropathy remains to be elucidated.

In this study, we applied the streptozotocin (STZ)-induced diabetic adrenomedullin gene knockout (AMKO) mice to clarify the role of adrenomedullin in the NADPH oxidase expression and its oxidative products in diabetic nephropathy, and we also investigated the relationship between adrenomedullin and the renin-angiotensin system that stimulates NADPH oxidase and oxidative stress.

## 2. Materials and Methods

### 2.1. Animals

The AMKO mice were generated in our laboratory as previously described [4]. The homoknockout of the adrenomedullin gene is embryonically lethal; therefore, we used the adrenomedullin gene heteroknockout mice in the experiments. The genotyping was performed with polymerase chain reaction (PCR) using the tail genomic DNA as a template and the +/+ littermates as wild controls. All mice were kept in a 12-hour light/12-hour dark room and fed mice pellets and tap water *ad libitum*. STZ at a dose of 50 mg/kg BW in citrate buffer (pH 4.5) was injected intraperitoneally at six weeks (*n* = 4 in each group) after 18 hours of fasting for five consecutive days. Four weeks after the last STZ injection, 24-hour urine samples were collected, and the mice were anesthetized with pentobarbital (5 mg/mouse). After abdominal incisions were created, blood samples were collected from the inferior vena cava, and the mice were then perfused with phosphate buffered saline (PBS) to wash out the blood, followed by 4% paraformaldehyde. The kidneys were excised and subjected to a histological examination. All procedures were performed in accordance with our university guidelines for animal handling.

### 2.2. Immunohistochemistry and Morphometry

The procedure for immunohistochemistry has been described previously [13, 17, 18]. Briefly, 2-*μ*m sections of kidney blocks were incubated with 3% H_2_O_2_ and blocking serum. The sections were incubated with a monoclonal antibody for NADPH oxidase component p47phox (Transduction Laboratories, Lexington, KY, USA) and a rabbit polyclonal antibody for malondialdehyde (MDA, Alpha Diagnostic International, San Antonio, TX, USA) at 1 : 100 dilution overnight. Sections were incubated with a biotinylated secondary antibody against mouse immunoglobulin for NADPH oxidase p47phox or with a biotinylated secondary antibody against rabbit immunoglobulin for MDA (Dako, Glostrup, Denmark) for two hours, followed by incubation with a horseradish peroxidase (HRP)-conjugated streptavidin solution. HRP labeling was detected using incubation with peroxidase substrate solution and diaminobenzidine (DAB, 0.8 mM, Dojindo Laboratories, Kumamoto, Japan). The sections were counterstained with hematoxylin. For a negative control, sections were processed in the same way without the primary antibody. Immunoreactivity for p47phox in the macula densa and for MDA in the glomerular mesangial area was scored as 0 for no staining, 1 for mild staining, 2 for moderate staining, and 3 for strong staining [18]. The scores from four animals were pooled in each group. The juxtaglomerular apparatus (JGA) was observed using periodic acid methenamine silver (PAM) staining. The presence of renin-secreting granular cells (GC) around the afferent arteriole was scored as 0 for no renin granules, 1 for few segmental cells containing renin granules in the JGA, 2 for all cells containing renin granules around the afferent arteriole, and 3 for renin-containing cells extended to the proximal portion of the afferent arteriole. The GC scores of four mice were pooled in each group. The correlation between the GC score in the JGA and the NADPH oxides expression score in the macula densa was calculated using a linear regression analysis. The degree of glomerular matrix expansion was scored in each glomerulus as previously described [19], and the correlation with the glomerular MDA immunostaining score was calculated using a linear regression analysis.

### 2.3. Western Blot for Prorenin

As described previously [13], the kidneys were homogenized, and samples containing 50 mg of protein were separated using SDS-polyacrylamide gel electrophoresis on a 4/20% gel and electroblotted on a nitrocellulose membrane. Western blotting was performed with rabbit anti-prorenin antibodies (Yanaihara Institute Inc., Shizuoka, Japan, 1 : 250 dilution) as the primary antibody and HRP-conjugated swine anti-rabbit immunoglobulin (Dako, Glostrup, Denmark) as the secondary antibody at 1 : 1,000 dilution. Next, the membrane was incubated with 0.8 mmol/L of diaminobenzidine (DAB; Dojindo Laboratories, Kumamoto, Japan) with 0.01% H_2_O_2_ and 3 mmol/L of NiCl_2_ to detect blots. The area and density of the bands of each stained protein were measured using the NIH image software package.

### 2.4. Measurements for Blood Glucose, Urinary Protein, and Plasma and Renal Angiotensin II

Blood glucose was measured by Glutest E II (Kyoto Dai-iti Kagaku, Kyoto, Japan), and urinary protein was measured by the Bradford method and corrected by urinary creatinine measured by Jaffe method using spectrophotometer [15]. Plasma and renal angiotensin II concentration was assessed by the radioimmunoassay method and corrected by the kidney weight [20].

### 2.5. Statistics

The values were expressed as the mean ± standard error. An analysis of variance was used for statistical comparisons among the four groups followed by a Bonferroni post hoc analysis. *P* values of less than 0.05 were considered to be statistically significant.

## 3. Results

### 3.1. Diabetes in AMKO Mice

Four weeks after STZ injection, the blood glucose levels significantly increased in both the wild DM mice and the AMKO-DM mice compared with those observed in the control wild and AMKO mice, and there were no differences in the blood glucose levels between the wild DM mice and the AMKO-DM mice (Table 1). The urinary protein excretion corrected for the level of urinary creatinine was significantly higher in the AMKO DM mice than in the wild DM mice (Table 1). It was impossible to measure the urinary protein levels of wild and AMKO control mice due to their extremely low urinary volume.

### 3.2. The NADPH Oxidase Expression and Lipid Peroxidation Products in the Kidneys

**Figure 1:**
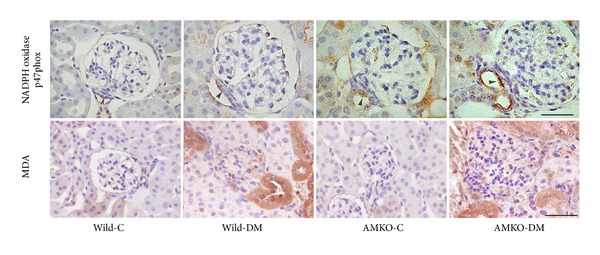
Immunohistochemistry of NADPH oxidase p47phox and malondialdehyde (MDA) in the kidneys of the control (C) and streptozotocin-induced diabetic (DM) in wild and adrenomedullin gene knockout (AMKO) mice. Immunoreactivity for p47phox and its product MDA was stronger in the glomeruli and distal tubules, including the macula densa (arrowhead), in the DM mice compared with that observed in the controls in both wild and AMKO mice, and was stronger in the AMKO-DM mice than in the wild-DM. The bar indicates 50 *μ*m.

Immunoreactivity for the NADPH oxidase p47phox in the glomerulus and distal tubules, including the macula densa, was increased in the wild-DM mice compared with that observed in the wild-control mice, and while the AMKO-DM mice exhibited further increases in NADPH oxidase p47phox compared with the AMKO-control mice (Figure 1). Associated with the changes in the NADPH oxidase expression, the lipid peroxidation products evaluated with MDA were increased in the glomeruli and distal tubules in both the wild-DM and AMKO-DM mice compared with that observed in each control, and the immunoreactivity for MDA was stronger in the AMKO-DM mice than in the wild-DM mice (Figure 1).

### 3.3. Glomerular Matrix Expansion Was Correlated with Lipid Peroxidation Products in Glomeruli

**Figure 2:**
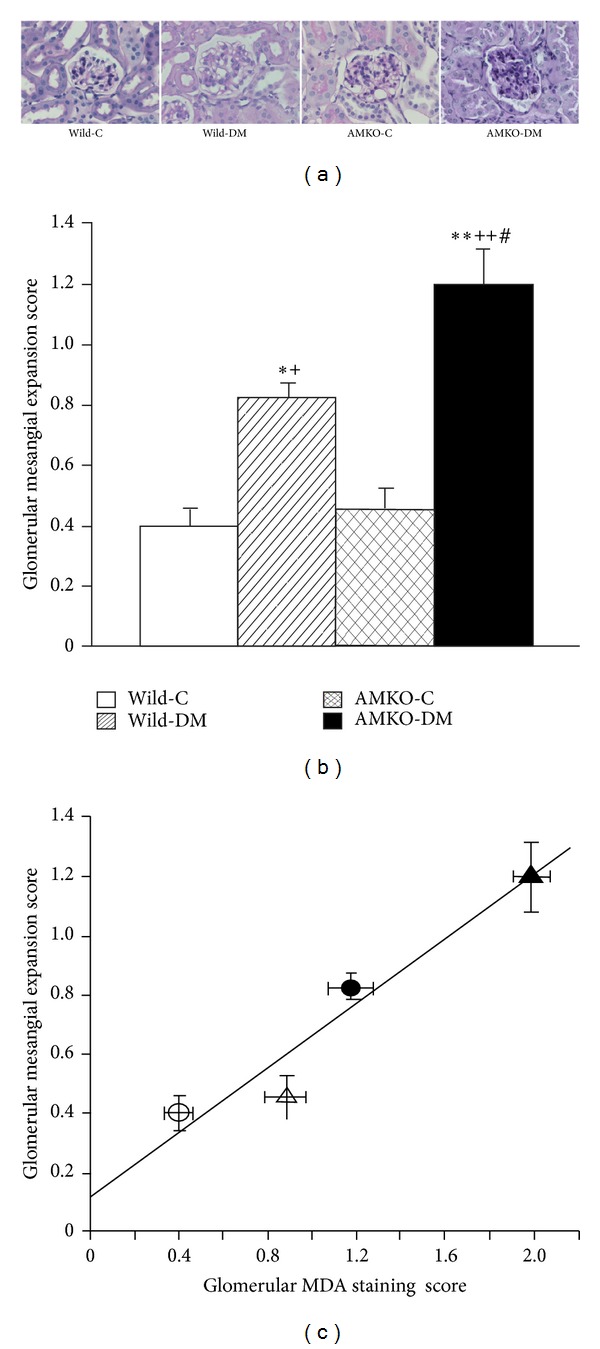
PAS staining of the glomerulus (a). The glomerular matrix expansion score (b) and its correlation with the glomerular malondialdehyde (MDA) staining score (c). The mesangial matrix was segmentally increased in both the STZ-induced diabetic wild mice (Wild-DM) and the diabetic adrenomedullin gene knockout (AMKO) mice (AMKO-DM) compared with that observed in the control mice (Wild-C and AMKO-C). (a) The bar indicates 50 *μ*m. (b) The AMKO-DM mice exhibited a significant increase in the mesangial matrix expansion score compared with the wild-DM mice. Four mice in each group. *P < 0.005, **P < 0.0001 versus Wild-C, ^+^ *P* < 0.01, ^++^ *P* < 0.0001 versus AMKO-C, and ^#^ *P* < 0.01 versus Wild-DM. (c) The white circles indicate wild-control mice, the black circles, diabetic wild mice, the white triangles indicate adrenomedullin gene knockout (AMKO)-control mice, and the black triangles indicate diabetic AMKO mice. *R* = 0.96, *P* < 0.05.

PAS staining demonstrated glomerular mesangial matrix expansion in the diabetic mice compared with that observed in the control wild and control AMKO mice (Figure 2(a)). The glomerular mesangial expansion scores were significantly higher in the diabetic mice than in the controls in both wild mice (0.83 ± 0.05 versus 0.40 ± 0.07, *P* < 0.005) and AMKO mice (1.20 ± 0.12, versus 0.46 ± 0.07, *P* < 0.0001), and the AMKO-DM mice exhibited further increases in mesangial matrix expansion than the wild-DM mice (*P* < 0.01, Figure 2(b)). There was a significant positive correlation between the glomerular MDA staining scores and the mesangial matrix expansion scores (Figure 2(c)).

### 3.4. Renin-Secreting Granular Cells and the NADPH Oxidase Expression

**Figure 3:**
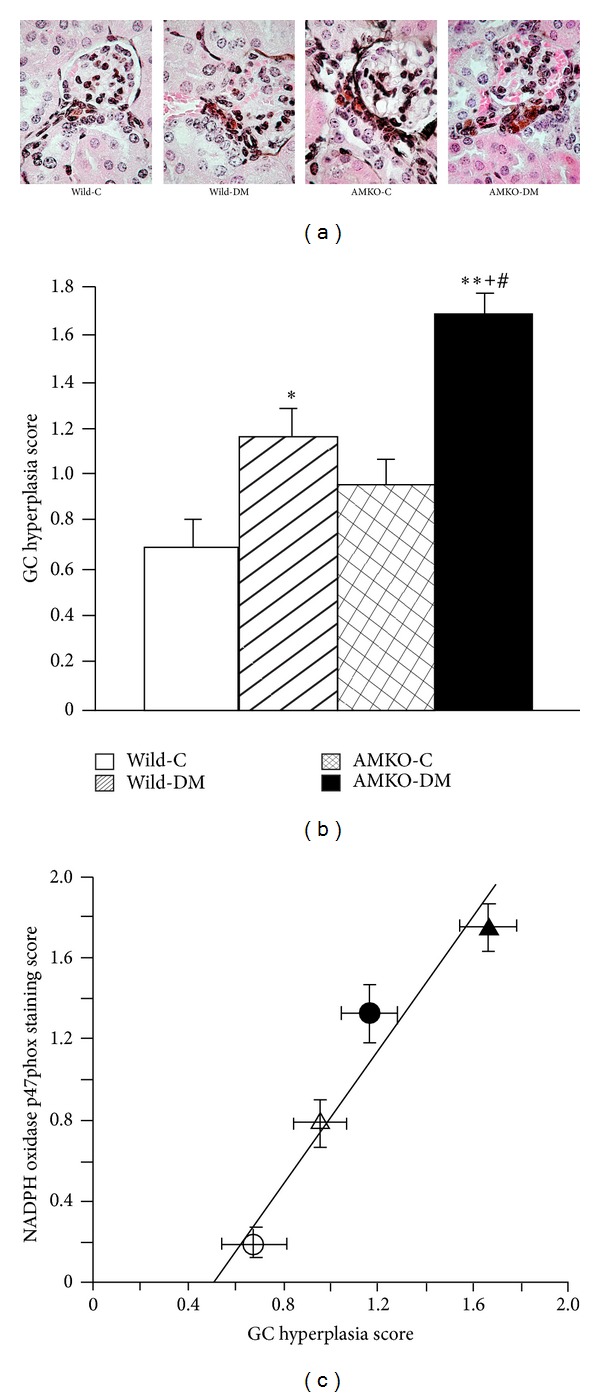
Renin-secreting granular cells in the juxtaglomerular apparatus and their correlation with the NADPH oxidase expression in the macula densa. (a) PAM staining illustrating granular cells in the juxtaglomerular apparatus with silver stained renin granules and reddish cytoplasm. The bar indicates 50 *μ*m. (b) The granular cell (GC) hyperplasia scores were increased in the STZ-induced diabetic wild mice (Wild-DM) and the adrenomedullin gene knockout (AMKO) mice (AMKO-DM) compared with those observed in the control mice (Wild-C and AMKO-C). The AMKO-DM mice exhibited further increases in granular cells compared with the Wild-DM mice. Four mice in each group. *P < 0.005, **P < 0.0001 versus Wild-C, ^+^ *P* < 0.001 versus AMKO-C, and ^#^ *P* < 0.01 versus Wild-DM. (c) The correlation between the renin-secreting granular cells (GC) hyperplasia score in the juxtaglomerular apparatus and the NADPH oxidase p47phox immunoreactivity score in the macula densa. The white circles indicate wild-control mice, the black circles indicate diabetic wild mice, the white triangles indicate adrenomedullin gene knockout (AMKO)-control mice, and the black triangles indicate diabetic AMKO mice. *R* = 0.96, *P* < 0.03.

**Figure 4:**
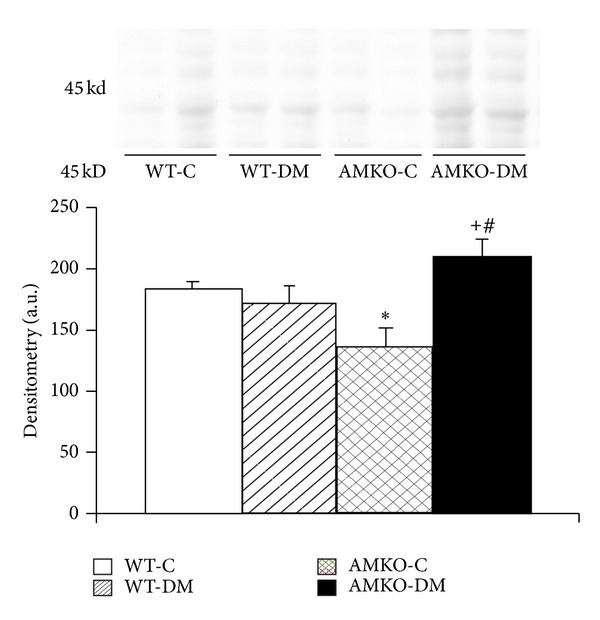
Western blotting of prorenin in the kidneys. The bands for prorenin are demonstrated at the molecular weight of 45 kD, and the densitometry of the bands is shown below (*n* = 4). Wild-C: wild-control mice, Wild-DM: wild mice with streptozotocin-induced diabetes, AMKO-C: adrenomedullin gene knockout (AMKO) mice without treatment (control), and AMKO-DM: AMKO mice with streptozotocin-induced diabetes. *P < 0.05 versus Wild-C, ^+^ *P* < 0.01 versus AMKO-C, and ^#^ *P* < 0.05 versus Wild-DM.

PAM staining identified the renin-secreting granular cells (GC) around the distal portion of the afferent arteriole in the juxtaglomerular apparatus with small granules (Figure 3(a)). The diabetic mice displayed GC hyperplasia compared with that observed in the controls in both wild (1.16 ± 0.12 versus 0.68 ± 0.14, *P* < 0.005) and AMKO mice (1.66 ± 0.12 versus 0.96 ± 0.11, *P* < 0.001), and the GC hyperplasia scores were significantly higher in the AMKO-DM mice than in the wild-DM mice (*P* < 0.01, Figure 3(b)). Western blotting of renal prorenin confirmed the increased renal prorenin production in AMKO-DM mice compared with that observed in the wild-DM mice (Figure 4). Plasma angiotensin II was significantly increased in AMKO-DM mice compared with that in wild-DM mice, and renal angiotensin II was significantly increased in AMKO-DM mice compared with that in AMKO-C mice. There was a significant positive correlation between the GC hyperplasia scores and the NADPH oxidase p47phox expression scores in the macula densa (Figure 3(c)).

## 4. Discussion

In the present study, we revealed that diabetic AMKO mice exhibit significant increases in the NADPH oxidase expression and lipid peroxidation product formation in the kidneys compared with diabetic wild mice. The presence of increased lipid peroxidation products in the glomeruli exhibited a positive correlation with the presence of mesangial matrix expansion. This indicates that endogenous adrenomedullin plays an important role in protecting against oxidative stress in the kidneys via the suppression of NADPH oxidase and can prevent glomerulosclerosis. This is consistent with our previous observation that the antioxidative stress action of adrenomedullin plays an important role in organ protection in cardiovascular disease [4, 6].

It has been shown that the production of adrenomedullin is increased in the vasculature in patients with diabetes [7, 8] and that the local action of adrenomedullin in the kidneys is upregulated in the early phase of diabetic nephropathy [9]. However, the role of adrenomedullin in diabetic nephropathy has not been clearly elucidated. To clarify the role of adrenomedullin in diabetic nephropathy, we induced diabetes using STZ in AMKO mice. We demonstrated that proteinuria and glomerular matrix expansion are more severe in the diabetic AMKO mice than in the diabetic wild mice. Therefore, we hypothesize that endogenous adrenomedullin in diabetes may act to protect against the development of diabetic nephropathy. This is supported by evidence showing that the genetic predisposition to develop diabetic nephropathy is associated with the microsatellite DNA polymorphism of the adrenomedullin gene [21].

Endogenous adrenomedullin plays a role in renoprotection by suppressing oxidative stress in the diabetic condition because we showed that oxidative stress production via NADPH oxidase is increased in diabetic adrenomedullin gene-deficient mice compared to that observed in diabetic wild type mice and that renal MDA production has a positive correlation with glomerular mesangial matrix expansion. Among several pathogenetic mechanisms of diabetic nephropathy including advanced glycated end-product formation, an enhanced PKC pathway, the polyol pathway, the hexosamine pathway, and the renin-angiotensin (RA) system [10], oxidative stress plays an important role in the development of diabetic nephropathy, while the suppression of oxidative stress ameliorates renal damage [12–15]. In this study, the NADPH oxidase expression in the glomeruli and distal tubules, including the macula densa, was increased in diabetic wild mice compared to that observed in control wild mice, confirming the findings of our previous study showing that the expression of p47phox, the regulatory component of NADPH oxidase, is increased in the kidneys of diabetic rats [13, 15]. The diabetic AMKO mice demonstrated further increases in the NADPH oxidase in glomeruli and distal tubules, indicating that endogenous adrenomedullin may suppress the NADPH oxidase expression and its oxidative products in the diabetic kidneys.

In contrast, oxidative stress can induce adrenomedullin production [1, 22, 23], and it is possible that adrenomedullin increases in patients with diabetes to counterbalance increased oxidative stress. Indeed, increased oxidative stress is associated with elevated plasma levels of adrenomedullin in hypertensive diabetic patients [24]. The induction of adrenomedullin by high levels of glucose is dependent on PKC activation [8], while PKC also activates NADPH oxidase via translocation of p47phox and p67phox to the membrane components and produces oxidative stress [25] that further increases adrenomedullin production. Moreover, the NADPH oxidase expression and renal MDA production did not show significant differences between the AMKO mice and the wild type mice in the control condition in the present study, suggesting that the role of endogenous adrenomedullin is not obvious in the control condition without stimuli to enhance oxidative stress. Based on our results, it is possible to assume that endogenous adrenomedullin exerts a negative feedback action on oxidative stress via the suppression of NADPH oxidase.

Hyperplasia of renin-secreting granular cells in the JGA indicates the activation of the renin-angiotensin system in the kidneys. Enhanced production of renal angiotensin II increases the mesangial matrix via TGF-*β* and is also well known to stimulate NADPH oxidase activity and radical production [26, 27]. In the present study, we showed that the scores of granular cell hyperplasia were higher in the diabetic animals compared with those observed in the controls in both wild and AMKO mice. This finding is consistent with those of previous reports showing that the JGA is enlarged in the early stage of type 1 diabetes [28, 29]. It has been reported that the level of local angiotensin II is increased in the kidneys of diabetes, although the circulating levels of renin are normal or even low in diabetic patients [30–33]. Interestingly, in this study, the diabetic AMKO mice exhibited an exaggerated enlargement of renin-secreting granular cells and renal prorenin compared with the diabetic wild mice. This suggests an interaction between adrenomedullin and the renin-angiotensin system. Some reports have demonstrated that adrenomedullin increases the plasma renin concentration [34] and also increases the release of renin from isolated perfused kidneys as well as from primary cultured granular cells [35]. On the other hand, chronic adrenomedullin administration in Dahl salt-sensitive rats inhibits increases in the plasma renin concentration, the aldosterone level, and the renal tissue angiotensin II levels [2]. Although renal angiotensin II level was suppressed in the AMKO-mice in the normal condition, our histological findings of increased granular cell hyperplasia and renal prorenin in AMKO-DM mice compared with those of Wild-DM mice and the findings of renal angiotensin II suppression in the chronic adrenomedullin-infused-Dahl salt sensitive rats raise the hypothesis that adrenomedullin counteracts the pathological activation of JGA and the renal renin-angiotensin system in diabetes and hypertension. In this study, there was a positive correlation between the granular cell hyperplasia score and the NADPH oxidase expression in the macula densa, indicating that increases in the renal renin-angiotensin system stimulate the NADPH oxidase expression in the kidneys of diabetic AMKO mice.

In conclusion, adrenomedullin gene knockout exaggerates diabetic mesangial matrix expansion and the NADPH oxidase expression and is associated with increased renin-secreting granular cell hyperplasia and renal prorenin. Therefore, we believe that endogenous adrenomedullin counteracts the pathogenesis of diabetic nephropathy possibly through an antioxidative stress action via the suppression of NADPH oxidase and the renin-angiotensin system. The activation of an endogenous adrenomedullin may therefore be a novel therapeutic approach for effective treating of diabetic nephropathy.

## Figures and Tables

**Table 1:** Physiological data.

	Wild-control	Wild-DM	AMKO-control	AMKO-DM
Body weight (g)	22.0 ± 0.4	16.9 ± 1.1^∗∗++^	24.5 ± 0.6	18.9 ± 0.9^∗++^
Blood glucose (mg/dL)	109 ± 3	361 ± 45^∗∗++^	161 ± 16	410 ± 27^∗∗++^
Urinary protein (mg/mg Cr)	ND	29 ± 3	ND	44 ± 4^#^
Plasma angiotensin II (pg/mL)	33.3 ± 11.0	19.0 ± 3.9	34.5 ± 1.5	59.3 ± 19.0^#^
Renal angiotensin II (pg/g kidney)	615 ± 102	685 ± 71^++^	269 ± 32*	458 ± 81^#^

*P < 0.05, **P < 0.001 versus Wild control, ^+^ *P* < 0.05, ^++^ *P* < 0.001 versus AMKO control, ^#^ *P* < 0.05 versus Wild-DM. *N* = 4 in each group.
